# Posterior short segment pedicle screw fixation and TLIF for the treatment of unstable thoracolumbar/lumbar fracture

**DOI:** 10.1186/1471-2474-15-40

**Published:** 2014-02-11

**Authors:** Ling Wang, Jianjun Li, Hong Wang, Qun Yang, Decheng Lv, Weiguo Zhang, Kai Tang, Limin Shang, Changming Jiang, Chunming Wu, Kai Ma, Bo Wang, Yang Liu, Rui Zhang, Xianping Shang, Depeng Kou, Xunyuan Jia, Xianglong Yang, Yilong Tang, Meng Zhang, Pengrui Wang, Yan Xu, Shijin Wang

**Affiliations:** 1 Department of Spine Surgery, First Affiliated Hospital of Dalian Medical University, Dalian 116011, People’s Republic of China; 2 Department of Oncology, First Affiliated Hospital of Dalian Medical University, Dalian, People’s Republic of China; 3 Department of Orthopaedics, Shengjing Hospital of China Medical University, Shenyang, People’s Republic of China; 4 Department of Orthopaedics, Armed Police Hospital of Dalian, Dalian 116013, People’s Republic of China

**Keywords:** Short segment fixation, Thoracic vertebrae, Lumbar vertebrae, Unstable burst fractures, Pedicle screw, TLIF

## Abstract

**Background:**

Currently, Posterior Short Segment Pedicle Screw Fixation is a popular procedure for treating unstable thoracolumbar/lumbar burst fracture. But progressive kyphosis and a high rate of hardware failure because of lack of the anterior column support remains a concern. The efficacy of different methods remains debatable and each technique has its advantages and disadvantages.

**Methods:**

A consecutive series of 20 patients with isolated thoracolumbar/lumbar burst fractures were treated by posterior short segment pedicle screw fixation and transforaminal thoracolumbar/lumbar interbody fusion (TLIF) between January 2005 and December 2007. All patients were followed up for a minimum of 2 years. Demographic data, neurologic status, anterior vertebral body heights, segmental Cobb angle and treatment-related complications were evaluated.

**Results:**

The mean operative time was 167 minutes (range, 150–220). Blood loss was 450 ~ 1200 ml, an average of 820 ml. All patients recovered with solid fusion of the intervertebral bone graft, without main complications like misplacement of the pedicle screw, nerve or vessel lesion or hard ware failure. The post-operative radiographs demonstrated a good fracture reduction and it was well maintained until the bone graft fusion. Neurological recovery of one to three Frankel grade was seen in 14 patients with partial neurological deficit, three grades of improvement was seen in one patient, two grades of improvement was observed in 6 patients and one grade of improvement was found in 6 patients. All the 6 patients with no paraplegia on admission remained neurological intact, and in one patient with Frankel D on admission no improvement was observed.

**Conclusion:**

Posterior short-segment pedicle fixation in conjunction with TLIF seems to be a feasible option in the management of selected thoracolumbar/lumbar burst fractures, thereby addressing all the three columns through a single approach with less trauma and good results.

## Background

It still remains controversial about the optimal management strategy for thoracolumbar/lumbar burst fracture [1-3]. Surgical treatment is generally recommended for patients with neurologic deficits or in those with severe instability. Currently, posterior short segment pedicle screw internal fixation is one of the most common operative approaches to treat unstable thoracolumbar/lumbar burst fracture. Although the clinical results of this surgery are usually satisfactory, progressive kyphosis and a high rate of hardware failure remain a concern. Lack of the anterior column supporting is the main cause of hardware failure [4-9]. Several procedures aiming to reinforce the anterior column have been introduced including transpedicular bone grafting, balloon-assisted vertebroplasty and corpectomy and cage placement to solve this problem. The efficacy of some methods remain debatable and each present technique has its advantages and disadvantages. We prospectively treated a consecutive series of 20 thoracolumbar/lumbar burst fractures with posterior short-segment pedicle screw fixation (that is, pedicle screw fixation one level cephalad to and one level caudad to a fracture) in conjunction with TLIF (transforaminal lumbar/thoracolumbar interbody fusion) to evaluate the feasibility and efficacy of this new technique.

## Methods

### Patients’ information and indications for surgery

This prospective study includes a consecutive series of 20 patients (15 males and 5 females) with acute traumatic thoracolumbar/lumbar fractures who were operated between January 2005 and December 2007 in our hospital by a single spine surgeon. All cases were fractures of type A3 according to the Association for the Study of Internal Fixation (AO) system [10,11].

The patients aged from 18 to 59 years (mean 38.4 years) and the indication for surgery were the presence of any one or more of the following: 1. presence of neurological involvement caused by the fracture or CT scanning of the affected level showed more than 50% spinal canal compromise; 2. more than 50% loss of anterior vertebral height [12-15] or local kyphosis angle exceeds 25 degree [12]. All patients were treated with the technique of posterior short-segment pedicle screw fixation in conjunction with TLIF.

In each case, plain X-ray film of the affected region and computed tomography scanning through the affected level was done to show fracture morphology before surgery and any potential complications related to placement of a cage and transpedicle screws. Magnetic resonance imaging was done before surgery to assess canal encroachment and for signal abnormalities in the spinal cord and other soft tissues (for e.g., indications of a tear in the interspinous or posterior longitudinal ligament).

All the patients underwent plain radiography in the early-postoperative period (1 week) and again at 3, 6, 9, 12 months after surgery and finally the last follow-up, but the postoperative CT and MRI were prescribed only for selected patients mostly because of financial reason.

Plain radiograph analysis included measurements of anterior body height and local kyphosis for determination of the severity of deformity.

**Figure 1:**
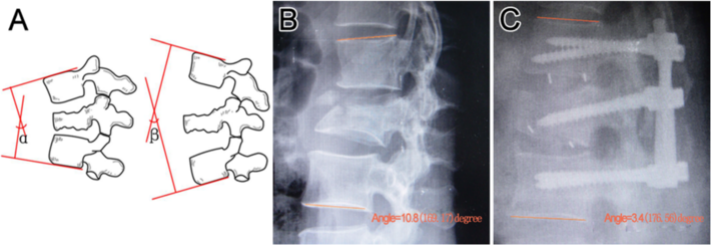
**Measurement of the kyphotic and lordosic angle. A** shows measurement of the angle of kyphotic deformity of the fractured segment was measured as the angle between the superior endplate of the vertebral body above the affected level and the inferior endplate of the vertebral body below the affected level, where kyphosis is recorded as a positive one (alpha), and lordosis is recorded as a negative one (gamma). **B** shows a kyphosis example (10.8°) and **C** shows a lordosis one (-3.4°) measured by the PACS measurement software.

The regional kyphotic angle (RA) of fractured segment was measured as the angle between the superior endplate of the upper adjacent vertebra and the inferior endplate of the lower adjacent vertebrae by the Cobb method (Figure 1) [16], where kyphosis is recorded as a positive one (alpha), and lordosis is recorded as a negative one (gamma).

**Figure 2:**
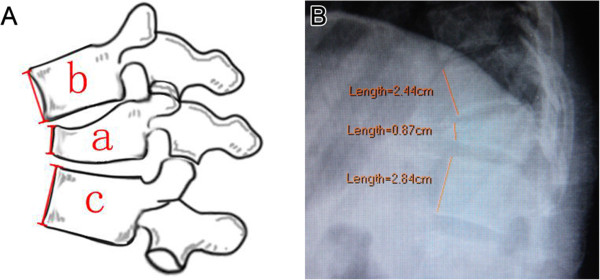
**Percentage of anterior body height compression (% ABC).** Percentage of anterior body height compression (% ABC) is calculated by the formula: % ABC = 100 - 2*a*/(*b* + *c*) 100, where a is the height of fractured vertebra; b is the height of the proximal vertebra; and c is the height of the distal vertebra **(A)**. **B** is an example measured by the PACS measurement software.

Anterior body height of the injured and the noninjured adjacent vertebrae above and below were measured on the lateral X-ray film, and the percentage of the fractured and restored anterior body height compression (% ABC) was calculated as the anterior height of the injured vertebra divided by the mean of the anterior height of the adjacent two vertebrae using the formula % ABC = 100 - 2*a*/(*b* + *c*) 100 adopted by Mumford et al. and Hak Sun Kim et al [17,18], where a is the anterior vertebral body height of fractured vertebra; b is the anterior vertebral body height of the proximal vertebra; and c is the anterior vertebral body height of the distal vertebra (Figure 2).

**Figure 3:**
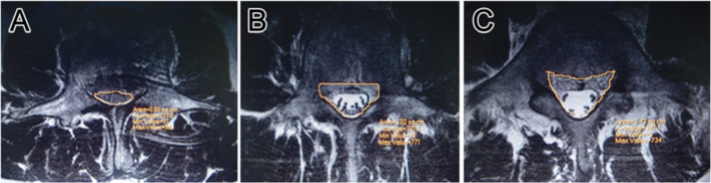
**Canal encroachment and the percentage of canal encroachment.** Canal area measured by the PACS measurement software. **A** is the area of the injured vertebra, **B** and **C** are that of the two adjacent vertebrae, and the percentage of canal encroachment (%CE) was calculated as the area of the protrusion into the canal of the injured vertebra divided by the mean of the maxim canal area of the adjacent two vertebrae, that is %CE = 100-2A/(B + C) × 100.

Canal encroachment was assessed by magnetic resonance imaging, and the percentage of canal encroachment was calculated as the area of the protrusion into the canal of the injured vertebra divided by the mean of the maxim canal area of the adjacent two vertebrae on axial magnetic resonance imaging of the injured segment. All the measurements were done using the PACS measurement software (Figure 3).

In addition, a fracture severity score was constructed by using the load-sharing classification (LSC) described by McCormack et al [19] which was measured by plain radiographs, CT scans to compare fracture severity.

For clinical assessment, neurologic deficit was assessed using Frankel motor score system [20]. Denis’ Pain and Work scales [21] were used to assess clinical outcomes. Associated lesions, intraoperative blood loss, operation time, hospital stay and complications were recorded for each case.

Research and publication utility of the operating data along with operative details were informed both in verbal and written notice to the patients. The participants included in this study were patients who provide a written informed consent which also authorized distribution of their individual information without giving a name. With the consent statement from the participants, we got ethical approval from the First Affiliated Hospital of Dalian Medical University Ethics Committee to conduct this prospective study.

### Surgical technique

The patient is placed in prone position on a radiolucent spine table. Apply manipulative reduction first if obvious kyphosis is detected. Fluoroscopy is used to locate the fractured vertebral body. A posterior midline straight incision centered on the affected level is made to expose the laminae 1 level above and below the affected level. Subperiosteal dissection is carried out with an electric cuter until the facet joints on both sides are visualized.

Pedicle screws are introduced 1 level below and above the affected level and also the fractured vertebral body [22,23] if the pedicles are intact and not expected to be removed for the purpose of decompression.

Spinal process and both lamina of the affected level are removed by rongeurs to decompress the posterior aspect of the thecal sac. Once posterior decompression was completed, the screws of both sides were distracted axially with contoured longitudinal rods to restore the segmental height and realign the spinal columns, which are verified by C-arm X-ray monitoring. Then the screws of more severe damaged side are released, and the ipsilateral facet joints are resected to reveal nerve roots. Epidural veins and radicular veins are cauterized with bipolar forceps to avoid massive bleeding. Dura mater is repaired if it is lacerated. Any adhesions between the posterior longitudinal ligament and the anterior surface of the thecal sac are released, thus, the thecal sac can be easily retracted to provide better exposure of the posterior portion of the vertebral body and the intervertebral discs. Then the thecal sac and nerve root are gently retracted and protected with a nerve retractor, and the adjacent intervertebral discs are completely removed.

The retropulsed fragment of the fractured vertebral body are hammered anteriorly back into the corpus using an ‘L’ angle dissector to recontour the posterior wall of the fractured vertebral body, at the same time decompressing the anterior aspect of the thecal sac.

Then granulated bone graft made from removed bone tissue is packed into the intervertebral space, some of the bone graft is packed into the vertebral body through the fractured endplate. Usually the autougenous bone is not enough and additional allograft bone is needed. The appropriate size of cage is confirmed by models and the cage is packed with granulated autougenous bone, then the cage is put into the intervertebral space and is positioned exactly at the midline. The same decompression procedure is done on the contralateral side if it is necessary, and before that the longitudinal rod is changed to the other side.

When the decompression procedure is finished by a recheck of all the neural elements involved, a second rod is placed and tightened. A final verification of the screws and cages positioning, alignment of the spinal columns and vertebral body height is done using posteroanterior and lateral fluoroscopy, then a drain is placed and the muscle, fascia and skin are closed in standard fashion.

### Follow-up data

During the postoperative period, walking and rehabilitation (in the rehabilitation center) were started as soon as the patients could withstand the pain and associated lesions of the patients permitted. The patients wore a corset for 12 weeks during the postoperative period, and heavy exercise was avoided within three months. All patients were evaluated according to clinical, radiologic, and functional parameters before surgery, one week after surgery, and the latest follow-up. The clinical parameters evaluated were the neurologic deficit according to Frankel motor score system, pain and work Scale of Denis.

### Statistical analysis

The data were analyzed by the paired t test and the Wilcoxon test, and the level of significance was set at 95% in all analyses.

## Results

Table 1 lists the demographic data, perioperative characteristics and follow-up data for each of the 20 patients (15 males and 5 females). The patients aged from 18 to 59 years (mean 38.4 years). The affected levels were T12 level in 5 patients, L1 in 2, L2 in 9, L3 in 3, and L4 in 1. All the cases were type A3 fracture according to the AO classification system, and there were 8 type A, 11 type B and 1 type E according to the Denis classification system [24]. The patients’ mean LSS points were 6.85 (Table 1).

**Table 1:** Demographics, perioperative characteristics and follow-up data of the patients

**No**	**Level**	**Gender/age**	**Injury (cause)**	**AO (type)**	**Denis (type)**	**LSC**	**Accompanying injuries**	**ISI (Days)**	**HS (Days)**	**%CE**	**Final pain scale (Dennis)**	**Final work scale (Dennis)**	**RA (°)**			**Neurology (Frankel)**		**% ABC**		
													**Preop**	**IMPO**	**Final**	**Preop**	**Final**	**Preop**	**IMPO**	**Final**
1	L4	M/47	TA	A3.3	A	8	Multiple rib fractures, emopneumothoras	14	25	87	P1	W1	2	-21	-20	C	D	48	7	8
2	T12	M/24	Fall	A3.1	B	7	Pelvic fracture	2	13	43	P1	W1	27	4	6	E	E	33	5	6
3	T12	M/25	IMPACT	A3.2	B	6		4	45	57	P1	W1	24	12	12	D	E	43	4	5
4	L3	M/45	Fall	A3.1	B	6	Calcaneus, talus	9	30	69	P1	W1	1	-12	-11	D	E	26	0	0
5	L1	F/40	TA	A3.3	A	7	Radius, ulna fracture	5	19	74	P1	W1	17	4	4	C	E	51	26	24
6	T12	M/18	Fall	A3.3	A	8	Abdominal injury	10	17	73	P1	W3	37	6	6	B	C	66	15	15
7	L2	F/33	Fall	A3.3	A	8		7	57	85	P1	w1	18	3	2	B	E	46	13	14
8	L2	M/34	Fall	A3.1	B	8	Calcaneus fracture	12	22	82	P1	w1	13	5	7	C	E	39	11	11
9	L2	F/41	TA	A3.1	B	7	Pelvic fracture	11	20	86	P1	W2	14	1	1	D	D	36	5	6
10	L1	M/56	TA	A3.1	B	6	Abdominal injury	9	25	63	P1	w1	19	10	9	E	E	37	5	5
11	L2	M/35	Fall	A3.1	B	6		5	15	59	P2	W2	1	-15	-18	E	E	18	1	0
12	L2	M/52	TA	A3.1	B	6		7	14	62	P1	W1	7	-12	-14	D	E	20	0	0
13	L3	M36	Fall	A3.2	A	7	Fabulas, talus fracture	1	34	79	P1	W1	22	6	4	C	E	45	11	12
14	L2	M/32	Fall	A3.1	B	5		6	20	61	P1	W1	25	4	7	E	E	26	0	1
15	T1	F/59	Fall	A3.3	A	7	Multiple rib multiple rib fractures, emopneumothoras	20	60	74	P1	W2	33	24	24	C	E	48	40	39
16	L2	M/45	Fall	A3.1	D	7	Radius fracture	6	34	75	P1	W1	17	-3	-2	C	E	34	3	6
17	L2	M36	TA	A3.3	A	7	Calcaneus fracture	9	22	65	P1	W1	6	-7	-7	D	E	44	7	6
18	L2	M35	Fall	A3.1	B	7	Coccal fracture	11	33	95	P1	W1	21	6	7	B	D	27	4	7
19	L3	F/30	TA	A3.3	A	8		9	23	87	P1	W1	2	-17	-16	E	E	57	12	14
20	T12	M/45	Fall	A3.2	B	6	Clavicle fracture	6	38	54	P1	W1	18	5	7	E	E	32	7	9

Abbreviations: *TA* traffic accident, *LSC* load sharing classification score, *ISI* Injury Surgery Interval, *HS* hospital stay, %*CE* percentage of canal encroachment, *RA* regional kyphotic angle, % *ABC* Percentage of anterior body height compression, *Preop* preoperaion, *IMPO* immediate postoperation, *Final* last follow-up.

Neurologic deficit was graded according to Frankel motor score system. Three patients were classified as Frankel B, 6 as Frankel C, 5 as Frankel D, 6 as Frankel E, there was no patient classified as Frankel A in this series. The causes of injury included 12 cases of falling, 7 cases of traffic accident, 1 cases of hitting by weights. 15 cases suffered from multiple injuries, including multiple rib fractures in 2 cases (both complicating emopneumothoras), 8 cases of limb fractures, 2 cases abdominal injury, 2 cases of pelvic fracture and 1 case of Coccyx fracture. The average Injury Surgery Interval was 8.2 days, ranging from 1 to 20 days. In patients with associated thoracic, abdominal, pelvic or extremity injury, the average interval increased to 12.5 days, ranging from 6 to 20.

All patients were treated with the technique of posterior short-segment pedicle screw fixation in conjunction with TLIF. Because the group was fully skilled and effective in treating degenerative spinal disease by TLIF, we didn’t encounter any difficulty in the operation process. The mean operation time was 167 minutes (range, 150–220). Average estimated blood loss was 820 ml (range, 450–1200). Average hospital stay (including hospital stay in the rehabilitation center) was 28.3 days, ranged from 13 to 60. In neurologically intact patients, average hospital stay decreased to 18.3 days, ranging from 13 to 25. The average follow-up was 29.5 months (range, 24–53).

Preoperative canal encroachment, preoperative and follow-up anterior vertebral body height, segmental Cobb angle and are listed in Table 1.

Neurological recovery of one to three Frankel grades was seen in 13 patients with partial neurological deficit, three grades of improvement happened in one patient (from grade B to grade E), two grades of improvement were observed in 6 patients and one grade of improvement was found in 6 patients. In only one patient with partial neurological deficit (Frankel grade D) on admission, no improvement was observed. All the neurological intact patients (6 cases) remained so during the follow-up period.

Local kyphosis was improved from 16.95° before surgery to 0.15° after surgery and progressed to 0.40° at the last follow-up. There was a significant difference between preoperative and postoperative values (P < 0.05) but no significant difference between the values of postoperation and last follow-up (*P* > 0.05). On an average, total kyphotic correction was 16.80°.

Preoperative average segmental height loss of the fractured level was 38.80%, postoperative value was 8.80%, and last follow-up value was 9.40%. There was a significant difference between preoperative and postoperative values (*P* < 0.05) and no significant difference between postoperative and last follow-up values (*P* > 0.05).

**Figure 4:**
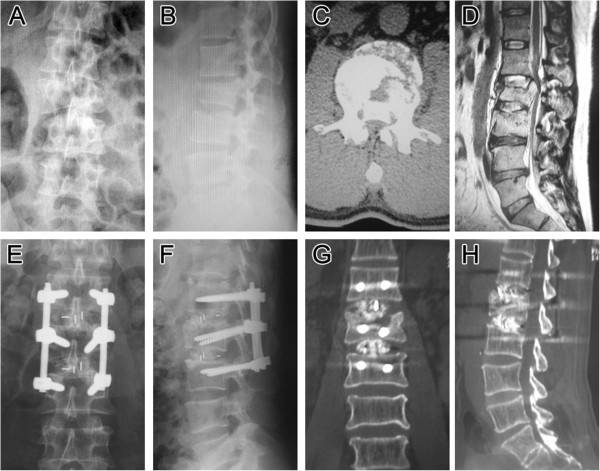
**Follow-up images (case 18).** Preoperative and postoperative imaging of a 45-year-old male patient (case 16) with L2 burst fracture, LSS _7, AO type A3.2, Danis type E with Frankel C neurologic impairment. **A**, **B**, Preoperative AP and lateral roentgenogram showing a L2 burst fracture with an angular segmental deformity of 17°. Preoperative axial CT scan image **(C)** and preoperative sagittal lumbar magnetic resonance imaging **(D)** demonstrating a significant spinal canal encroachment by retropulsion of the fragments of the fracture vertebra with laminar fracture. **E**, **F**, Postoperative AP and lateral plain radiograph showing a correct position of two cages placed to intervertebral space and the posterior transpedicular system. Angular deformity was corrected and segmental height was restored. **G**, **H**, Postoperative axial and sagital CT demonstrating a significant spinal canal clearance and satisfied position of the cages and normal thoracolumbar alignment and some of the grafted bone chips was inserted into the vertebral body through the fractured endplate.

**Figure 5:**
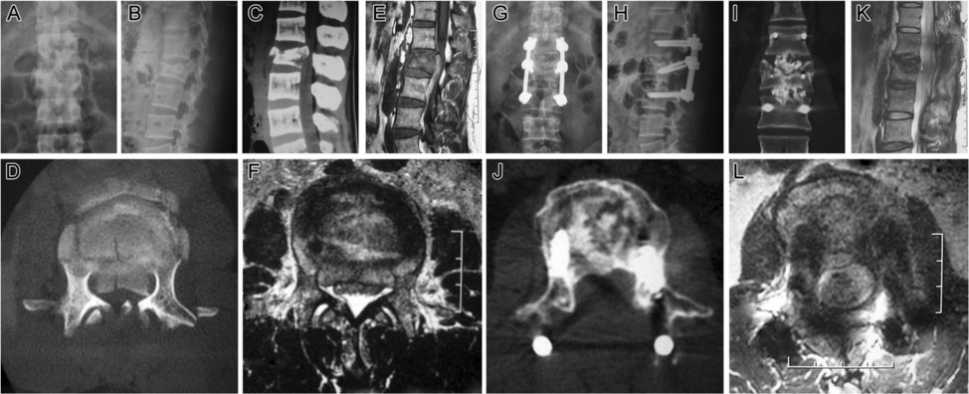
**Follow-up images (case 18).** Preoperative and postoperative imaging of a 35-year-old male patient (case 18) with L2 burst fracture, LSS _7, AO type A3.3, Danis type A with Frankel B neurologic impairment. **A**, **B**, Preoperative AP and lateral roentgenogram showing a L2 burst fracture with an angular segmental deformity of 21°. Preoperative sagittal **(C)** and axial **(D)** CT scan image, preoperative sagittal **(E)** and axial **(F)** lumbar MRI demonstrating a significant spinal canal encroachment at the fracture level. **G**, **H**, nine months postoperative AP and lateral plain radiograph showing a correct position of two cages placed to intervertebral space and the posterior transpedicular system. Angular deformity was corrected and segmental height was restored. **I**, 9 months postoperative coronal CT demonstrating successful intersomatic bony fusion of the L1 to L3 segment. **J**, 9 months postoperative axial CT demonstrating significant spinal canal clearance and remodeling of the posterior wall of the fractured vertebra body. **K**, **L**, 9 months postoperative sagittal and axial MRI showing satisfied decompression of the neural eliments.

Preoperative average canal encroachment was 71.50%, and in the 5 patients who undertook CT (Figure 4) and /or MRI (Figure 5) during follow-up period, the canal encroachment was 0 to 5%.

All patients recovered with solid fusion of the intervertebral bone graft (Figure 5), without main complications like misplacement of the Pedicle screw, infection, nerve or vessel lesion or hard ware failure. Two patients experienced cerebrospinal fluid leakage because of initial injury.

At the last follow-up, 95% patients complained of no pain (Denis Pain Scale P1), only one patient experienced occasional minimal pain with no need for medication (Denis Pain Scale P2). Most of the patients (80%) returned to previous employment (Denis Work Scale W1) at the last follow-up, 15% of the patients returned to previous employment but with labor restrictions (Denis Work Scale W2), only one case (5%) was unable to return to previous employment but working full time at a new job (Denis Work Scale W3).

## Discussion

Burst fractures of thoracolumbar/lumbar spine can cause neurologic complications and kyphotic deformity, [24-26] which may have a great impact on the patients’ life quality. Disputes about the best treatment for spine fracture among doctors as there are, it has come to a consensus among most scholars that operation is needed to treat unstable thoracolumbar/lumbar burst fractures and fractures with existing or potential nerve handicap. Biomechanical and clinical studies have shown that when there is loss of more than 50% of the vertebral body height or more than 25° angulation deformity of the injured segment, acute spinal instability results, and the spinal segment will eventually fail with weight-bearing [27]. Here we present a consecutive series of 20 patients with acute traumatic thoracolumbar/lumbar unstable burst fractures who need to be operated on according to the above criteria.

Evolution in spine hardware and surgical technique has offered an ample variety of instrumentation and surgical approaches. Common surgical options include anterior approach decompression and reconstruction, posterior pedicle screw fixation, and combined anterior-posterior approach [19,28-32].

Each technique has advantages and disadvantages [4,33]. No ideal surgical approach exist at present. By anterior approach we can decompress nerves sufficiently and provide reliable anterior column support. However, this approach requires longer operation duration and the rate of approach related complication and the death rate is significantly higher than posterior approach. Although the combination of anterior and posterior approach can provide the most stable biomechanical repair, the operation time, complication and morbidity rate might be apparently higher than that of the single approach. Traditionally the standalone posterior approach is relatively an easy procedure but can only indirectly reduce a fractured vertebral body, and the means of augmenting the anterior column are limited [34,35]. Reported loss of reduction caused by insufficient anterior column support with or without hardware failure is not uncommon [30,36,37].

Posterior approach instrumentation can be devided into long-segment fixation (involving more than two upper and lower neighboring levels), short-segment fixation (involving one level above and one below the fractured level) and mono-segment fixation. Nowadays short-segment pedicle screw instrumentation is a well described and popular technique to reduce and stabilize thoracic and lumbar spine fractures [38]. Short-segment fixation offers the advantage of saving motion segments when compared with longer instrumentations. On the contrary, investigators in recent studies have reported earlier implant failure and correction loss as the most important disadvantages of this method [5,7,39,40]. Controversy still exists about whether short-segment pedicle screw instrumentation is a suitable method for unstable thoracolumbar/lumbar burst fracture.

Several anterior column augmentation procedures have been offered as alternative solutions to prevent failure.5 Transpedicular grafting of the injured anterior vertebral body in addition to short-segment fixation has been introduced as a possible solution by Daniaux *et al*[41] in 1986. In the following years the benefits of transpedicular intracorporeal grafting has been supported by many researchers as well [42-45]. On the contrary several other groups, however, have reported disappointing follow-up results like high rate of reduction loss and implant failure and argued against the effectiveness of transpedicular grafting of the fractured vertebrae with short-segment fixation [30,36]. The large bone defect created inside the fractured vertebra after height restoration has been speculated to be the most important cause of these complications. While other studies have emphasized a loss of correction after implant removal due to the collapse of the intervertebral disc into the vertebral body through the fractured Endplate [9,46].

Thereafter another technique in which an anterior reinforcement is performed by bone cement through the posterior approach is described. The use of cement offers immediate stability but it does not correspond to a proper bone fusion and thus long term fate is unknown. Its use is reserved to specific indications because, in cases of severe posterior wall breakage the risk of cement leakage or bone fragment repulsion is high [47], and in practice, it is hard to anatomically reduce the fractured endplate and maintain the reduction till the fracture union, and this will finally result in loss of correction caused by intervertebral disc collapse.

Recently, Ayberk *et al*[48], Sasani and Ozer [49] and Yang Haiyun *et al*[50] have reported pedicle screw fixation combined with vertebra body reconstruction using the posterior approach for thoracolumbar burst fracture, which has been described by Tomita *et al.* for spinal tumor resection. By this TRSP (Three-Column Reconstruction Through Single Posterior Approach) technique the anterior and middle column is reconstructed by expandable or nonexpandable cage or mesh filled with autograft bone chips after a subtotal corpectomy of the fractured vertebral body. TRSP can decompress and repair neural elements circomferancialy and provide 3-column stabilization mechanically at the same time. Anterior cage can significantly decrease the load over the posterior fixation system and avoid the loosening or fatigue fracture of hardware,36,37 reduce the loss of the interbody height, prevent secondary kyphotic deformity, and the reported clinical outcomes were satisfied. Meanwhile TRSP is technically demanding, the learning curve is relatively long, and surgical trauma of this procedure is relatively large with more intraoperative and postoperative blood loss and more time of operation.

In 1991 Daniaux et al [51] suggest that transpedicular discectomy and intercorporeal grafting of the upper injured disc space may be more effective compared with intracorporeal grafting alone, based on the assumption that a substantial amount of correction loss occurs at the injured disc space.

In recent years, the application of transforaminal interbody fusion (TLIF) in spinal degenerative disease is becoming more and more common with sound results [52]. Huang RC and his colleagues practiced TLIF on a 24-year-old female patient with old T11-12 Chance fracture and successfully repositioned 38º kyphosis and achieved reliable interbody fusion and complete pain relief [53]. Here, we modified the Daniaux technique to combine TLIF with posterior short-segment pedicle screw fixation and investigate its feasibility of treating unstable thoracolumbar/lumbar burst fractures that surgical treatment is recommended and achieved favorable immediate outcome. Such literature has not been reported.

Once again the techniques that we used in this paper is not the same as the original concept of TLIF, but because the whole process is just like TLIF, so we still name it TLIF, and it is the main point of innovation from our team. In some cases we did observed that the cages sinked into the vertebral body when they were inserted into the disc space more or less, but the patients in this case series wore a long hard corset for 12 weeks during the postoperative period, and heavy exercise was avoided within three months, and at the final follow up, no cages migration was observed in this case series. In our operation design, the cage inserted in the disc space with endplate broken served mostly as a large bone block to keep the autograft particles in place, and partly as an anterior and middle column strut.

With the help of intact ALL and annulus it is easy to reduce the fracture by posterior pedicle screw devices, but how to deal with the large amount of bone defect inside the fractured vertebral body? Instead of a subtotal corpectomy of the fractured vertebral body and replace it with mesh or cage in TRSP technique, we try to reduce the protruding fragments of the posterior wall by hammering them anteriorly back into the fractured vertebra body, and with the constraint of intact ALL and annulus, intracorporeal and intercorporeal grafting becomes very safe, eliminating the worry about bone chip migration especially after cage insertion into the intervertebral space, which will make the bone graft contained in a relative closed space.

Because TLIF technique avoids the excessive spinal cord and nerve root traction, significantly reduces the risk of nerve damage, and removal of articular process and even pedicle of the fractured vertebral body makes detection of the anterior wall of the canal and repositioning of the protruding fragments easy and safe. Pedicle screw devices allow immediate stable fixation as the screws traverse all the three columns, and by impact autograft into the intervertebral space and even into the vertebral body through the fractured endplate after disc excision, and cage insertion into the intervertebral space, we reconstruct the anterior and middle columns of spine and provide additional immediate stability. Together with immobilization by postoperative external fixation, we achieve sound satisfactory effect with less operative trauma and less blood loss. And because most of the blood supply of the fracture fragments is left intact, the fracture union duration might be theoretically shortened to a certain degree.

At the beginning of our study of this new technique, we fixed with polyaxial pedicle screws for the above and low adjacent vertebrae, and reduction loss happened in most cases, that were not included in our series. We used monoaxial pedicle screws to fix the above and low adjacent vertebrae for all the cases presented here, in which reduction was well maintained till bony union without hardware failure. So we suggest using monoaxial pedicle screws to fix the above and low adjacent vertebrae and polyaxial pedicle screws for the fractured one, it makes it convenient for the installation of the longitudinal rod and avoids the reduction loss.

We treated AO type A3 fractures in our series by posterior short segment pedicle screw fixation and TLIF, but did not include the AO type B or C fractures, because we are not certain about whether the stability provided by our technique is sufficient for fractures mechanically caused by apparent distraction and/or tortional forces, which might need more segment pedicle screw fixation. We are now plan to explore the feasibility of this new technique for the treatment of AO type B or C fractures in the near future.

In summary, posterior short segment pedicle screw fixation and TLIF might be an acceptable option that owns relative merits at present for selected thoracolumbar/lumbar burst fractures, especially for AO type A3, Denis type A, B, C or E fractures and for low lumbar fractures where incomplete contact between the cage and the endplate exists because of apparent lordosis [48] and the main blood vessels make anterior approach more difficult or in patients with anterior approach contraindications like abdominal injury, but more patients and further observation need to be carried out.

## Conclusion

Posterior short segment pedicle screw fixation and TLIF might be an optimal surgical treatment option for selected unstable thoracolumbar/lumbar burst fractures, especially in fractures of AO type A3, Denis type A or Denis type B, C and E with pedicle fracture. Most spinal surgeons are familiar to TLIF, and the technique itself allows easy detection of the anterior wall of the canal and safely repositioning of the protruding fragments, avoiding excessive spinal cord and nerve root traction, thus significantly reduces the risk of nerve damage. Anterior and middle columns reconstruction by intracorporeal grafting together with intercorporeal grafting especially cage insertion can provide enough additional biomechanical stability until solid bony union of the fractured fragment without reduction loss. And because most of the blood supply of the fractured vertebral body is left intact, the intraoperative blood loss is decreased and the fracture union duration might be theoretically shortened to a certain degree. However, the authors didn’t suggest by this study that posterior short segment pedicle screw fixation and TLIF is the best choice for the treatment unstable thoracolumbar/lumbar fracture, we only tried to provide an additional option. The most suitable treatment depends on the fracture classification and the patient’s condition. The small patient number and lack of a comparison group limit the value of this study. Further investigation need to be carried out to evaluate the effect of this new technique in highly unstable thoracolumbar/lumbar fractures like AO type B or C.

### Key points

• To assess the effect of a new technique, posterior short segment pedicle screw fixation and TLIF for the treatment of selected unstable thoracolumbar/lumbar fracture.

• Anterior and middle columns reconstruction by intracorporeal grafting together with intercorporeal grafting especially cage insertion can provide enough additional biomechanical stability until solid bony union.

• TLIF technique avoids the excessive spinal cord and nerve root traction, significantly reduces the risk of nerve damage.

• Posterior short segment pedicle screw fixation and TLIF is relatively less invasive as compared with the present techniques, and most of the blood supply of the fracture fragments is left intact, the fracture union duration might be theoretically shortened to a certain degree.

• Posterior short segment pedicle screw fixation and TLIF might be an optimal option for the treatment of selected unstable thoracolumbar/lumbar fracture, especially AO type A3, Denis type A,B,C or E fractures. Further investigation need to be carried out to evaluate the effect of this new technique in highly unstable thoracolumbar/lumbar fractures like AO type B or C.

## Competing interests

The authors confirm that there are no known conflicts of interest associated with this publication and there has been no significant financial support for this work that could have influenced its outcome.

## Authors’ contributions

All authors contributed to the design, conduct, analysis and/or interpretation of the investigation reported herein. All authors participated in the preparation, review and approval of this article. CJ, CW, KM, BW, YL, and RZ contribute to the surgery, and KT, LS contribute to the data collection.

## Authors’ information

Ling Wang and Jianjun Li are the joined first co-author.

## Pre-publication history

The pre-publication history for this paper can be accessed here:

http://www.biomedcentral.com/1471-2474/15/40/prepub
